# Liquid Biopsy: A New Translational Diagnostic and Monitoring Tool for Musculoskeletal Tumors

**DOI:** 10.3390/ijms222111526

**Published:** 2021-10-26

**Authors:** Argyris C. Hadjimichael, Alexandros Pergaris, Angelos Kaspiris, Athanasios F. Foukas, Stamatios E. Theocharis

**Affiliations:** 1 First Department of Pathology, Medical School, National and Kapodistrian University of Athens, 75, Mikras Asias Street, Bld 10, Goudi, 11527 Athens, Greece; ortho.argiris@gmail.com (A.C.H.); alexperg@yahoo.com (A.P.); 2 Third Department of Orthopaedic Surgery, “KAT” General Hospital of Athens, Nikis 2, 14561 Kifissia, Greece; afoukas1@otenet.gr; 3 Division for Orthopaedic Research, Laboratory of Molecular Pharmacology, School of Health Sciences, University of Patras, 26504 Patras, Greece; angkaspiris@hotmail.com

**Keywords:** liquid biopsy, soft tissue sarcomas, bone sarcomas, biomarkers, cancer, diagnosis, prognosis, treatment

## Abstract

Soft tissue and bone sarcomas represent a group of aggressive neoplasms often accompanied by dismal patient prognosis, especially when distant metastases are present. Moreover, effective treatment can pose a challenge, as recurrences are frequent and almost half of patients present with advanced disease. Researchers have unveiled the molecular abnormalities implicated in sarcomas’ carcinogenesis, paving the way for novel treatment strategies based on each individual tumor’s characteristics. Therefore, the development of new techniques aiding in early disease detection and tumor molecular profiling is imperative. Liquid biopsy refers to the sampling and analysis of patients’ fluids, such as blood, to identify tumor biomarkers, through a variety of methods, including qRT-PCR, qPCR, droplet digital PCR, magnetic microbeads and digital PCR. Assessment of circulating tumor cells (CTCs), circulating free DNA (ctDNA), micro RNAs (miRs), long non-coding RNAs (lncRNAs), exosomes and exosome–associated proteins can yield a plethora of information on tumor molecular signature, histologic type and disease stage. In addition, the minimal invasiveness of the procedure renders possible its wide application in the clinical setting, and, therefore, the early detection of the presence of tumors. In this review of the literature, we gathered information on biomarkers assessed through liquid biopsy in soft tissue and bone sarcoma patients and we present the information they can yield for each individual tumor type.

## 1. Introduction

### 1.1. Soft Tissue and Bone Sarcomas

Soft tissue and bone sarcomas represent a group of generally aggressive neoplasms, arising from cells of mesenchymal origin. It is estimated that 13,460 people will be diagnosed with soft tissue cancer in 2021, as approximately 0.4 percent of men and women will develop one during their lifetime, while 5-year survival amounts to 65% [1]. With approximately 40% of patients presenting with metastasis at the time of diagnosis and advanced disease bearing a significantly grimmer outcome [1], the development of novel methods aiding in timely tumor detection remains of utmost importance. Moreover, early diagnosis of disease recurrence is imperative, as 5-year survival in cases with distant metastasis drops to 15% [1]. As far as bone sarcomas are concerned, chondrosarcoma represents the most common primary bone tumor in adults, comprising 40% of primary bone sarcomas, followed by osteosarcoma (28%), chordoma (10%) and Ewing sarcoma (8%) [2]. Like their soft tissue counterparts, bone sarcomas are accompanied by substantially poorer prognosis when disease is advanced. For chondrosarcoma, 5-year survival drops to 22% when metastasis is present, in contrast to 91% 5-year survival rates of localized disease [2]. The immense impact of timely tumor detection has directed the medical community towards the development of methods that identify the presence of neoplastic disease at early stages. Serum biomarkers are already utilized routinely in clinical practice for soft tissue and bone tumor prognosis and monitoring. To name but a few, alkaline phosphatase (ALP) and lactate dehydrogenase (LDH) in osteosarcoma and myoglobin in rhabdomyosarcoma are used as diagnostic and prognostic biomarkers. However, sensitivity and specificity of ALP at the time of tumor detection amounted to 53.2% and 90.1% respectively. Moreover, in patients with metastasis, sensitivity remained at 53.2% but specificity dropped at 78.2% [3]. Such results indicate the necessity for further research on the discovery of novel biomarkers that will effectively contribute to the fields of tumor detection and monitoring. Latest research has unveiled the molecular mechanisms responsible for tumorigenesis in many mesenchymal neoplasms, paving, thus, the way for the development of specialized treatment regimens. In Gastrointestinal Stromal Tumors (GISTs), identification of c-kit mutation led to targeted treatment with imatinib, yielding impressive results regarding patients’ survival. In non-GIST soft tissue sarcomas that harbor PDGFR gene mutation, as well as in 10% of GISTs that exhibit this specific mutation, the PDGFR–targeting drug olaratumab has been approved for treatment [4]. Such characteristic examples only represent a small part of the ongoing advances in specialized molecular therapies targeting genetic abnormalities present in soft tumor cancer cells. However, they underline the need for novel, efficient and inexpensive methods that reveal the molecular signature of each individual tumor, aiding in the development of a specialized treatment regimen, as well as patients’ prognosis estimation.

### 1.2. Liquid Biopsy

The term liquid biopsy refers to the sampling and analysis of patients’ fluids, such as blood, to identify tumor biomarkers. “Biomarkers” comprise of a broad-spectrum of biological molecules which are indicators of normal and pathogenic processes and could be either upregulated or downregulated after a response to a clinical intervention (e.g., surgical excision, chemotherapy, radiotherapy) [5]. Conventional technical approaches adopted to investigate plasma-derived biomarkers such as immunohistochemistry (IHC), fluorescence in situ hybridization (FISH) and Enzyme-linked Immunosorbent Assay (ELISA) are nowadays being replaced with high-throughput techniques due to the application of modern technological capacities [6]. Parameters assessed through novel liquid-biopsy techniques include, among others, circulating tumor cells (CTCs), circulating free DNA (ctDNA), micro RNAs (miRs), long non-coding RNAs (lncRNAs), exosomes and exosome–associated proteins [7]. A wide range of methods has been utilized by researchers, such as qRT-PCR, qPCR, droplet digital PCR, magnetic microbeads and digital PCR [7]. Tumor cells, as well as tumor cells undergoing apoptosis and necrosis, release ctDNA and miRs in blood circulation. Moreover, DNA can be extracted from CTCs, as well as from circulating exosomes. Therefore, it becomes apparent that information regarding the genetic alterations of a tumor can be obtained [8]. DNA abnormalities, such as amplifications, deletions and translocations, chromosomal abnormalities and point mutations can be detected, revealing the molecular profile of the tumor [8]. The techniques utilized are minimally invasive and can be proven to be exceptionally useful in cases where the location of the tumor renders the histological evaluation difficult. Consequently, timely diagnosis, assessment of the primary site, progression and detection of recurrences can be achieved [8]. By capturing the molecular landscape of a tumor, as well as detecting possible molecular changes in tumor cells, personalized treatment regimens can be developed that will uttermost benefit patients’ outcome. Moreover, aggressive tumor types and potential drug resistance can be assessed through molecular profiling of tumor cells [9]. Lastly, treatment monitoring through measurement of blood levels of certain biomarkers, which can exhibit stability or increase in cases of treatment resistance, can prove a useful tool in the clinical setting [10]. A major challenge regarding the pre- clinical aspects for ideal liquid biopsy application in clinical specimens requires the establishment of blood sample handling protocols accompanied by reliable processes for the isolation of circulating tumor DNA (ctDNA). According to Gerber et al. blood collection in EDTA tubes for a maximum of 4 h in a room temperature or for up to 24 h when stored at 4 °C is highly recommended to guarantee a great quality of genomic analyses for the liquid biopsy [11].

**Figure 1:**
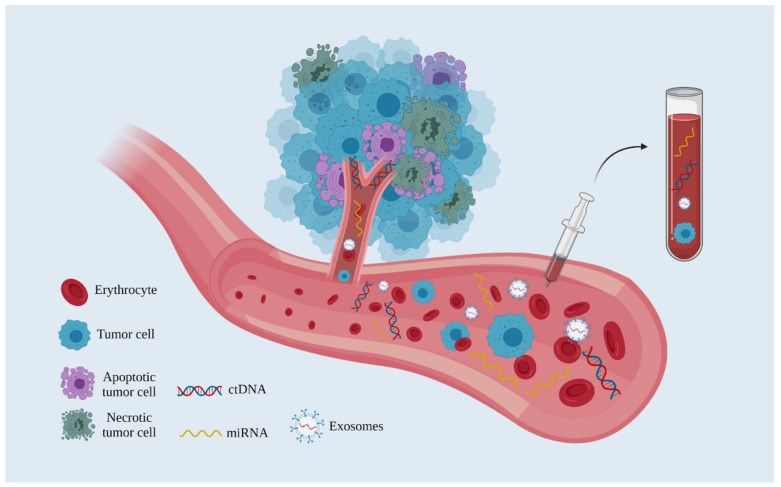
The liquid biopsy technique. Note that in addition to apoptotic and necrotic tumor cells, viable tumor cells also release DNA and miRNAs in the blood circulation (Created with BioRender.com, accessed on 21 October 2021).

The liquid biopsy technique is illustrated in Figure 1.

### 1.3. Research Strategy

The authors explored appropriate studies addressing the liquid biopsy technique in order to investigate the role of different biomarkers as diagnostic and prognostic tools in musculoskeletal tumors. A systematic computer-based literature review search with predefined criteria was performed in the following databases: MEDLINE/PubMed (1946–present) of the National Library of Medicine and EMBASE (1947–present). The research methodology used a combination of the following terms: “liquid biopsy [All fields]”, “soft tissue sarcomas [All fields]”, “bone sarcomas [All fields]”, “biomarkers [All fields]”, “cancer [All fields]”, “diagnosis [All fields]”, “prognosis [All fields]”, “treatment [All fields]”. Searching of the reference lists of potentially relevant origin was also performed. The electronic literature search was conducted independently by two authors (A.C.H., A.P.). Moreover, the senior authors (A.K., A.F.F. and S.E.T.) independently screened the titles and abstracts to identify relevant studies evaluating the application of liquid biopsy in musculoskeletal tumors.

### 1.4. Inclusion Criteria and Study Selection

Inclusion criteria were: papers written in English, peer-reviewed journals and clinical studies concerning the application of liquid biopsy in the clinical setting of musculoskeletal oncology. In addition, in vitro and in vivo studies were eligible for inclusion that evaluated the implication of the liquid biopsy technique on the early detection and monitoring of soft tissue and bone sarcomas.

Exclusion criteria were: articles using language other than English, letters to the editor, expert opinion publications, surveys with insufficient details about the correlation between the application of liquid biopsy with monitoring and clinical outcome of patients suffering from soft tissue and bone sarcomas.

### 1.5. Data Extracted

Articles that conformed to the above-mentioned criteria were retrieved and all of the studies related to these were extensively searched. The senior authors (A.K., A.F.F. and S.E.T.) examined all the identified surveys, extracting data using a predetermined form. All data about the type of biomarkers, the samples and methods used in liquid biopsy from each study were extracted and summarized in Table 1 and Table 2 for soft tissue and bone sarcomas, respectively. The two authors (A.C.H., A.P.) extracted data from reviewed studies about the genetic alterations in each tumor type and the biomarkers detected for each tumor in liquid biopsy and results were presented in the figure which is included in our article. The presence of duplicate studies was examined using the Endnote software (Clarivate Analytics, Philadelphia, PA, USA).

## 2. Soft Tissue Sarcomas

### 2.1. Liposarcoma (LPS)

LPS is the most common soft tissue sarcoma in adults, as it accounts for about 20% of all mesenchymal malignancies [39]. Most frequently, LPSs arise from deep soft tissues of lower limbs (24% of all limb sarcomas) and retroperitoneum (45% of all retroperitoneal sarcomas) [40]. LPS is classified in 3 subtypes according to WHO, based on the morphological characteristics of each group: well-differentiated/dedifferentiated liposarcoma (WDL/DDL), myxoid/round-cell liposarcoma (MRCL) and pleomorphic liposarcoma (PLS) [41].

These subtypes differ from each, based on the specific genetic alterations they exhibit, which could play a potential role in liquid biopsy for tumor monitoring. The WDL/DDL type can be distinguished by amplification of the chromosomal region12q13-15, which contains oncogenes like MDM2, CDK4 and HMGA2 [12]. The MRCL type includes the t (12;16) (q13; p11) translocation, that generates the FUS-DDIT3 (FUS-CHOP) fusion protein [12]. Gits CM et al. supported that the microRNA expression profiles can distinguish the liposarcoma subtypes. According to their published data in biopsy samples, miR-145 and miR-451 act as tumor suppressors in adipose tissue and could be evaluated in liquid biopsy as promising biomarkers for monitoring either the recurrence or the progression of LPS [12]. A different study examined the monitoring value of the breakpoint t (12:16) and TERT C228T mutation in liquid biopsy of plasma samples of patients with myxoid liposarcoma. The t (12;16) and TERT C228T ctDNA were detected prior to surgical excision of the tumor and were significantly reduced after tumor removal and during lack of recurrence during follow-up [13]. Liquid biopsy was obtained from 21 patients with DDL, who were enrolled in a cohort study which evaluated the therapeutic value of SAR405838 (inhibitor of the HDM2–p53) in tumor suppression. The TP53 mutations appearing in circulating cell-free DNA (cfDNA) were found to be a reliable monitoring tool for DDL expansion [14].

The role of miR-3613-3p as a specific potential biomarker was identified through liquid biopsy from blood samples of DDL patients. Using the qRT-PCR technique, they observed significantly upregulated (fold change: >2.5; *p* < 0.05) levels of miR-3613-3p in patients with dedifferentiated liposarcoma (*n* = 6) compared to healthy individuals (*n* = 4) [15].

### 2.2. Leiomyosarcoma (LMS)

LMS is a soft tissue sarcoma derived from smooth muscle cells that can be subdivided into three groups of origin: uterine, abdominal (retroperitoneum and gastro-intestinal) and non-visceral. They can arise from extremities, representing approximately 2–10% of all soft tissue tumors, as well as from bones in even rarer instances [42]. The most frequently affected patients belong to the age group between 50–70. In the majority of cases, the tumor has a sporadic distribution, but it can be also developed in patients who received radiation therapy or suffer from a hereditary disease, like retinoblastoma or Li-Fraumeni syndrome [43]. It has been shown that the loss of tumor-suppressor genes, including RB1, p53 and PTEN, can be the substrate for leiomyosarcoma to develop, although mutations in single-nucleotides have not yet been identified [44].

Hemming et al. evaluated the utility of circulating tumor DNA (ctDNA) as a blood-based biomarker in patients with leiomyosarcoma to detect progression or relapse of disease using the non-invasive liquid biopsy technique [16]. In 11 (69%) out of 16 patients who showed LMS progression and a primary tumor >5 cm in diameter, the ctDNA was detectable. Additionally, in 16 patients with low grade disease, the liquid biopsy revealed no amounts of circulating ctDNA, in contrast with detection of high levels of ctDNA in patients with increasing tumor size [16]. Subsequently, the ctDNA was characterized as a remarkable biomarker in LMS, as it diminished with tumor resection but increased in tumor relapse.

A more recent study by Demoret et al., in concordance with that of Hemming ML, revealed that ctDNA was present in liquid biopsy platforms of 6/6 patient who have been suffering from LMS [17]. Despite of prior reports, their results suggested that ctDNA was not a reliable biomarker in monitoring LMS, as the blood levels of ctDNA were associated with neither radiographic finding of tumor volume expansion nor the number of metastatic sites [17].

### 2.3. Ewing Sarcoma (ES)

ES is the second most prevalent osseous and soft tissue malignancy in childhood after osteosarcoma. It is identified as a highly aggressive neoplasm which belongs to the group of small round cell tumors [45]. The median age of diagnosis is 15 years and the predominant origin of primary tumors are the pelvis (25%), femur (16%), ribs (13%), spine (8%), and scapula (5%) [45]. It is reported that 25% of newly diagnosed patients suffer from metastatic disease, mostly in their lungs and bones [46].

In the majority of ES cases (85%), the EWS-FLI1 (11;22) (q24; q12) gene translocations arise from the fusion of ES breakpoint region 1 (EWSR1) with the transactivation domain of FLI1, which belongs to the E26 transformation-specific (ETS) family of transcription factors [47]. In 10% of all ES cases the translocation involves fusions between EWS and other ETS family members such as ERG, FEV and ETV1, which interfere with tumorigenic pathways, including cell growth, proliferation and differentiation [47].

The role of exosomes as biomarkers in the detection of ES progression was evaluated in a preclinical setting by extracting extracellular vesicles (EVs) from ES cell-lines. Increased levels of the exosome-associated proteins CD63 and CD81 were observed through flow-cytometry and electron microscopy [18]. Subsequently, the use of qRT-PCR was proposed in order to amplify the sensitive ES-specific transcript EWS-FLI1 for detection of residual disease from peripheral blood samples of patients with ES [18]. Tsugita et al. conducted further in vitro and in vivo trials to examine the efficacy of microvesicles as possible diagnostic markers in ES patients [19]. Regarding their results, the microvesicles containing the EWS/FlI1 mRNA were detected from the medium of cultured ES cells carrying the translocation (11;22) (q24; 12) [19]. Additionally, the analysis of aspirated blood samples from xenograft nude mice who were injected with TC135 or A673 EC cells were found to be quite remarkable. The EWS/Fli-1 mRNAs were measurable by quantitative PCR in up to 40% (2/5 mice) of blood samples, suggesting that the detection of microvesicles could be a non-invasive technique for monitoring the evolvement of ES [19].

The detection of circulating tumor cells in blood samples of eighteen ES patients was demonstrated by Benini et al. using the liquid biopsy technique [20]. The isolation of ES tumor cells was achieved by targeting their CD99 surface markers through immunoseparation with CD99-antibody and magnetic microbeads. Circulating tumor cells were found above the limit of 1 cell/mL in blood samples of ES patients but not in blood samples of healthy donors [20]. Using the RT-qPCR technique for detection of ES gene transcripts, 11 patients were EWSR1/FLI1 type 1- positive, five patients were EWSR1/FLI1 type 2-positive, one patient was EWSR1/ERG type 1- positive, and one was EWSR1/ERG type 9e–positive [20].

The kinetics of EWSR1 fusion sequence was evaluated in 234 plasma samples of 20 ES patients [21]. Nineteen patients had the EWSR1-FLI1 fusion gene and one patient the EWSR1-ERG fusion sequence. The application of chemotherapy in ES patients reduced the tumor volume, as depicted in CT and MRI scans, and diminished the circulating EWSR1 fusion sequence levels measured via droplet digital PCR. Larger local or metastatic tumors exhibited higher blood levels of EWSR1 fusion transcripts. Additionally, the commence of chemotherapy induced the fast reduction of circulating cell-free tumor DNA (ctDNA), which returned in high levels in patients with tumor recurrence [21]. Allegretti et al. observed that the EWS-FL1 rearrangements can be directly identified using RT-qPCR from ES tumor tissues extracted RNAs. Additionally, they used digital PCR (dPCR) to amplify ctRNAs from plasma specimens by the noninvasive liquid biopsy technique [22]. They concluded that ctRNA levels were correlated (*p* < 0.05) with tumor’s progression parameters, such as the metabolic volume and the total lesion glycolysis, which were identified by positron emission tomography (PET) [22]. The ctRNA levels were measured through liquid biopsy and were recognized as a fundamental monitoring tool for the ES progression as well as for the disease’s response during the surgical and chemotherapeutic treatment [22].

Liquid biopsy has a remarkable role in monitoring the tumor burden in ES patients utilizing a highly personalized technique. The tumor specific EWS-ETS fusion gene breakpoint plasma tumor DNA fragments can be detected by droplet digital PCR (ddPCR) method, as presented by Hayashi et al. in 2016. The blood analysis of 3 ES patients revealed that measurable levels of plasma tumor DNA, following resection of primary tumors and administration of chemotherapy, could be an alert sign indicating tumor relapse, while disease is still clinically and radiographically undetectable [23].

The most recent study in 2020 by Samuel et al. evaluated the effect of 619 proteins composing the subset of ES derived small extracellular vesicles as biomarkers in monitoring the tumor burden at diagnosis, responsiveness to therapy and recurrence [24]. The CD99/MIC2 and the NGFR membrane proteins were chosen to develop a liquid-based assay in order to detect the mRNA cargo of well-established transcripts in ES, such as the EWS-ETS. The liquid biopsy from ES pediatric patients with localized or metastatic disease showed/revealed that the identification of novel extracellular vesicle proteomics has significant diagnostic value [AUC = 0.92, *p* value = 0.001 for vesicles numeration, with a Positive predictive value = 1.00, 95% CI = (0.63, 1.00) and a Negative predictive value = 0.67, 95% CI = (0.30, 0.93)] compared with tumor-free donors [24].

### 2.4. Rhabdomyosarcoma (RMS)

RMS represents a high-grade sarcoma of skeletal myoblast-like cell origin [48]. The incidence of RMS amounts to 4.9 patients per million people aged <15 years, indicating RMS as the most common pediatric soft tissue sarcoma [49,50]. RMS is further classified in three subtypes: the alveolar RMS (ARMS), the embryonal RMS (ERMS), and the pleomorphic rhabdomyosarcoma (PRMS), which develop through different molecular mechanisms.

The well-known specific gene translocations in ARMS are the t(1;13 (p36;q15), which results in the PAX3/FOXO1 fused genes, as well as the t(2;13)(q35;q14) translocation, that generates the PAX7/FOXO1 fusion [25,51]. Sorensen PH et al. demonstrated, in 171 pediatric ARMS individuals, that the expression of PAX3/FOXO1 and PAX7/FOXO1 translocations induce a significantly worse outcome compared to patients without them, due to a higher risk for metastatic disease [51]. They further recorded a better overall survival in PAX7/FOXO1 positive patients (4-year overall survival was 77%) compared to PAX3/FOXO1 positive ones (4-year overall survival was 52%, *p* = 0.08), based on the primary tumor evaluation [51]. The t(1;13)(p36;q15) and t(2;13)(q35;q14) translocations are not usually detectable in ERMS and PRMS patients, but the PAX3, PAX7 and FOXO1 genes are frequently overexpressed [51]. In our opinion future studies would be able to evaluate the prognostic role of the above fusion transcripts in RMS using liquid biopsy technique.

According to an experimental in vitro trial of Miyachi et al., the expression of muscle-specific miRNAs (miR-1, -133a, -133b and -206) in RMS human cell lines was significantly higher compared with their expression in non-RMS cultures [26]. Importantly, the serum levels of the specific miR-206 in RMS patients were significantly elevated compared to serum levels of non-RMS patients, demonstrating an important correlation with in vitro findings. Additionally, analysis of plasma samples of RMS patients after treatment revealed that the expression levels of each of the muscle-specific miRNAs were remarkably decreased compared with non-treated ones [26]. Muscle-specific miRNAs, especially miR-206, could become a reliable biomarker for RMS progression via the non-invasive liquid biopsy analysis.

Another group of researchers used the ultralow passage whole-genome sequencing technique to investigate the role of liquid biopsy technology in the detection of ctDNA in 7 patients with ARMS [25]. Their study showed that ctDNA was measurable in ≥ 50% of pre-treatment plasma samples from patients with ARMS. All patients with ctDNA specific blood sampled were positive for PAX3/FOXO1 translocation, indicating the promising role of liquid biopsy in RMS’s progress evaluation [25].

### 2.5. Synovial Sarcoma (SS)

SS is a malignant and very aggressive mesenchymal tumor which accounts approximately for 10% of all soft tissue sarcomas [52]. It mostly affects pediatric populations and young adults with a peak incidence in the third decade of life [52]. Although extremities are the most common locations to develop, SS could evolve at any site of the body [52]. The pathognomonic abnormality for SS is the genetic translocation t(X;18((p11.2q11.2)), resulting in the fusion specific transcripts SS18-SSX1, SS18-SSX2 and rarely the SS18-SSX4, in >95% of cases [52,53]. The presence of SS18-SSX fusion transcripts in either fresh tumors or paraffin-embedded biopsy tissues can be detected by conventional RT-PCR, qRT-PCR and dual color FISH techniques [54]. However, the function of SS18-SSX oncoproteins and their association with the pathogenetic mechanisms which provoke the expansion and progression of synovial sarcoma remain controversial [55,56].

A study conducted by Mihály et al. attempted to explore the prognostic role of SS18-SSX fusion genes in 15 patients with synovial sarcoma by liquid biopsy [27]. The droplet digital PCR and the nested PCR techniques were utilized in order to amplify SS18-SSX fusion genes from circulating tumor cells and cell-free nucleic acids [27]. At the time of diagnosis, 10 patients had metastatic disease and 5 localized tumors. Based on their findings, the SS18/SSX2 fusion transcript was only detected in small amounts in only one patient by droplet digital PCR. Consequently, the detection of the SS18-SSX fusion gene after surgical excision and/or chemotherapy/radiotherapy was infrequent and failed to monitor the tumor recurrence. These results confirm previously published data that quantification method by droplet digital PCR shows 10-fold greater sensitivity compared with RT-qPCR [57]. In agreement with the aforementioned report, a larger study by Przybyl et al. incorporated 38 patients with synovial sarcoma and revealed that nested RT- PCR had a 5.3% sensitivity and 100% specificity in detection of SS18-SSX1/2 fusion transcript [28]. Another previously conducted study also by Przybyl et al. had already shown that only 9% (3/34) of patients diagnosed with SS had measurable circulating tumor cells in their blood. The low detection of SS18-SSX1/2 in circulation depicted a diminished role of this marker in the monitoring of synovial sarcoma’s progression [58].

A recent study by Uotani et al. examined the effectiveness of circulating miRNAs as fluid biomarkers for the detection of patients suffering for synovial sarcoma and their prognostic role in tumor dynamics [29]. Their findings demonstrated that serum levels of miR-92b-3p were significantly higher in patients with synovial sarcoma compared to healthy individuals [area under the curve -AUC of 0.77 (95% confidence interval = 0.61–0.94)] and patients diagnosed with other soft tissue sarcomas [AUC was 0.88 (95% confidence interval = 0.72–1.0)] [29]. They further observed that cell-free miR-92b-3p was stable and was contained within exosome fractions, rather than bound to the protein Argonaute-2, which plays a fundamental role in the RNA silencing processes. Moreover, the measured blood levels of miR-92b-3p were associated with the stages of tumor progression in transplanted mice with the human synovial sarcoma cell line SYO-1, indicating its prognostic role in this neoplasm [29].

Hashimoto et al. examined the biopsy specimen from a 22-year-old pregnant woman with synovial sarcoma in the thigh. Using RT-PCR, they observed the expression of the specific SYT-SSX fusion gene transcript of synovial sarcoma in the neoplasm [56]. Due to pregnancy, the administration of chemotherapy was postponed, and a wide local resection of the tumor was performed to the patient’s thigh [56]. Seven weeks later, multiple pulmonary metastases developed, and the patient received 3 cycles of chemotherapy (doxorubicin, cyclophosphamide, ifosfamide) post-delivery. Blood samples from the mother, child and placenta were evaluated for the presence of circulating SYT-SSX fusion gene by nested PCR. A 212bp product representing the SYT-SSX fusion gene was extracted from circulating tumor cells. While it was measurable in woman’s blood before the removal of the tumor, it was undetectable post-surgery [56]. The results of nested PCR from fetal and placenta’s blood were in accordance with RT-PCR analysis of the tissue samples. Tumor cells were not widespread in the fetus and the circulating levels of SYT-SSX fusion gene proved to be of prognostic value regarding the progression of the synovial sarcoma of the woman [56].

Table 1 summarizes the biomarkers investigated for soft tissue tumors detection and monitoring, the number of patients included in the studies, the methods utilized and the results.

**Figure 2:**
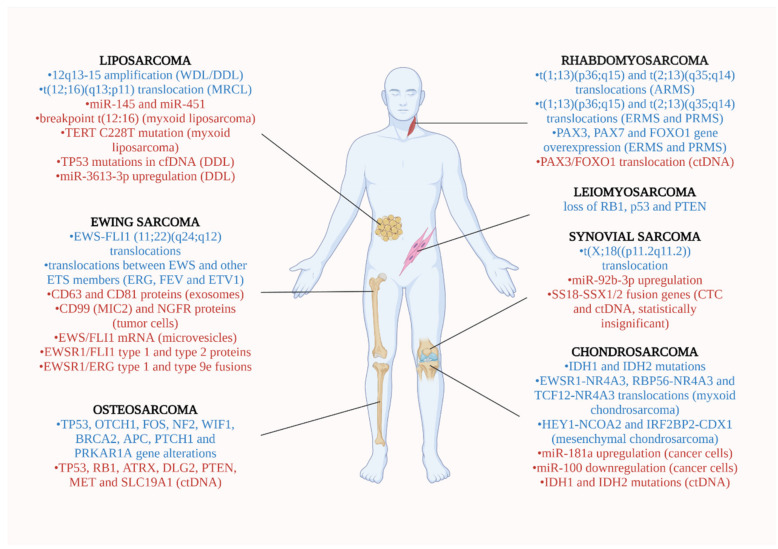
Soft tissue and bone sarcomas’ most common locations. Blue fonts: genetic alterations identified in each tumor type. Red fonts: Biomarkers detected for each tumor in liquid biopsy (Created with BioRender.com, accessed on 21 October 2021).

The most common sites of occurrence for each soft tumor, the genetic alterations identified in each tumor and the biomarkers detected in liquid biopsy are presented in Figure 2.

## 3. Bone Sarcomas

### 3.1. Osteosarcoma (OS)

OS is the most common primary malignant osseous tumor overall, which mostly arises within the metaphysis of long tubular bones of upper and lower extremities and rarely from flat bones [59]. OS is usually diagnosed in the 2nd–3rd decades of life and its prognosis depends on the presence of metastatic disease. Approximately, one quarter of patients present with pulmonary metastases at the time of initial diagnosis [59]. Recent studies in understanding the pathogenesis of OS revealed that no genetic translocation is associated with the tumor expansion. Therefore, numerous chromosomal abnormalities and mutations are involved in OS formation with *TP53* being the most frequently altered gene [60]. Laboratory markers, such as ALP (alkaline phosphatase) and LDH (lactate dehydrogenase) are usually interpreted in OS patients, albeit, it has been shown that their serum concentrations lack specificity and sensitivity and they are not always predictive of disease progression and response to treatment [61]. Furthermore, the bone resorption markers: tP1NP (total procollagen type 1 amino-terminal propetide) and β-CTx (β-isomerized C-terminal telopeptides) were found elevated in OS patients compared to healthy volunteers. However, they have not been recognized as reliable markers as their blood levels were irregular and not indicative of tumor expansion clinically [62]. Considered the need for a more reliable and non-invasive method for diagnosis and monitoring of tumor progression, liquid biopsy represents a potential monitoring tool in OS.

A cohort study by Shulman et al. examined the levels of ctDNA in banked plasma samples of 72 newly diagnosed OS patients, using next-generation sequencing (NGS) assays [30]. Forty-one out of seventy-two patients (56.9%) had detectable ctDNA in their blood samples at the time of diagnosis. Therefore, the authors observed a weak correlation between total cell-free DNA and the percentage of ctDNA, as only 11% (range 4.6–58%) of total cell-free DNA was ctDNA in patients who had detectable ctDNA (56.9%). Additionally, a higher risk for worse outcome and death was significantly associated with increased levels of ctDNA but not with total cell-free DNA [30]. Furthermore, the 8q gain was detected in 74.4% (32 out of 43) OS patients who were ctDNA positive. The 3-year free survival for patients with the 8q gain detected in ctDNA was found to be 60.0% (95% CI, 40.5–75.0) compared to 80.9 (95% CI, 42.4–94.9) in those without it (*p* = 0.18) [30]. Barris et al. [31] performed a targeted Next-Generation Sequencing (NGS) analysis to detect mutated alterations in TP53, RB1, ATRX, DLG2, PTEN, MET, and SLC19A1 genes in primary tumor samples as well as in ctDNA collected from blood samples of 7 OS patients at various time points (4 weeks prior-treatment of primary tumor-134 weeks post-treatment). According to the findings, clinical relapse was noted among three patients with germline-tumor pairs. In addition, single nucleotide variants (SNVs) were found in the tumor DNA of two patients with tumor relapse, but not in their genomic DNA. However, the small sample size was a limitation of the study and conclusions about the prognostic role of ctDNA in OS progression were not able to be extracted reliably [31].

Currently, liquid biopsy can be performed in patients suffering from OS to detect circulating levels of miRNAs. MiRNAs are non-coding RNA molecules with the ability to interfere with DNA and regulate gene expression. Consequently, miRNAs function as tumor-mediators, acting either as suppressors or enhancers of cancer development [63]. It has been shown that the downregulation of tumor suppressors miRNAs in OS patients (e.g., miR-326, miR-133b, miR-206, miR-152, miR-95–3p, miR-34b, miR-195, miR-223, miR-497) decreases apoptosis and increases proliferation, invasiveness and metastasis of tumor cells, promoting, therefore, systematic disease [32]. On the contrary, the overexpression of oncogenic miRNAs (e.g., miR-21, miR-196a, miR-196b, miR-195–5p and miR-199a-3p, miR-199a-5p, miR-221, miR-27a) has been correlated with cancer progression, end-stage disease, distant metastases and poorer prognosis. Subsequently, miRNAs have exhibited a promising potential value as serum biomarkers for detection and clinical evaluation of OS patients during follow-up as well as for assessment of therapeutic interventions and responsiveness to chemotherapy [32].

Similarly, to serum miRNAs, another type of gene regulators called Long Non-Coding RNAs (LncRNAs) play a significant role in OS monitoring and can be detected in blood via Liquid Biopsy techniques. Notably, the overexpression of a plethora of LncRNAs like Taurine up-regulated gene 1 (TUG1), 91H (interacts withH19/insulin-like growth factor 2), UCA1 (urothelial carcinoma associated 1), LncRNA activated by transforming growth factor-β (lncRNA-ATB) and metastasis associated lung adenocarcinoma transcript 1 (MALAT1) were found significantly elevated in OS patients compared with their healthy counterparts [32]. Interestingly, increased circulating levels of each of these biomarkers in OS patients were significantly correlated with systemic expansion, tumor relapse and poorer prognosis [32].

To date, the isolation and detection of circulating tumor cells (CTCs) has become a reliable method of monitoring the metastatic status of OS patients. Quantification of CTCs is a very challenging technique which distinguishes tumorous from normal circulating cells through specific molecules overexpressed only in CTCs [64]. CTCs of OS express the surface-type Vimentin (intermediate filament protein) due to their mesenchymal origin and can be discriminated from mononuclear blood cells which express the intracellular-type. Thus, the circulating blood levels of CTCs act as predictive and prognostic biomarkers for OS progression [33]. A recent study evaluated the expression of ezrin (a membrane-cytoskeleton linker protein) in CTCs by RNA probe technology. According to the results, the high expression of ezrin in CTCs correlated significantly with the formation of distant metastases (χ^2^ = 152.51, *p* = 0.000) in end-stage IIIB (Enneking staging system) disease compared with free of metastases early-stage disease (IIB) [34]. Another study applied a novel method for quantification of CTCs by assessing aneuploidy (abnormal chromosome numbers instead of surface markers) using the fluorescence in situ hybridization (FISH) technique to detect the centromeres of chromosome 8 [35]. Patients with pulmonary metastases revealed higher circulating levels of CTCs compared with patients free of metastases. In addition, high CTCs levels were correlated with poorer prognosis for OS patients [34].

The detection of serum extracellular vesicles is a promising useful tool for diagnosis and monitoring of OS patients. These nanovesicles carry important information within mRNAs, miRNAs and proteins in the bloodstream and modify the microenvironment of metastatic sites to welcome and support circulating OS cells [65]. Likewise, they reflect biological changes of OS cells which are the producing cells and determine the progression of tumor as well as the response to chemotherapy [65]. The in vivo study by Baglio et al., showed that OS cells from mice xenograft models were able to secrete extracellular vesicle (EV)-educated mesenchymal stem cells (TEMSC) which expressed a membrane-associated form of TGFβ. The overexpression of TGFβ induced the production of IL-6. The researchers showed that TEMSCs promoted tumor progression, while the administration of tocilizumab, a pharmaceutical inhibitor of IL6 and TGFβ, restricted the expansion of OS [36]. Moreover, they demonstrated that circulating levels of EV-associated TGFβ were useful monitoring biomarkers in OS as they were found increased in OS patients [36]. A good chemotherapeutic response could be predicted in OS patients by monitoring exosomes that transport specific miRNAs. It has been shown that reduced levels of transported miR-124, miR133a, miR-199a-3p, and miR-385 were significantly correlated with poor chemotherapeutic response in OS patients compared to good responders [37]. Subsequently, exosomal miRNAs could be reliable circulating biomarkers to discriminate poor from good responders, leading to elective changes in the therapeutic approach in some of OS patients.

### 3.2. Chondrosarcoma (CS)

CS is the second most common primary bone tumor which forms cartilaginous neoplastic tissue. Treating CS can prove quite challenging due to its resistance to both chemotherapy and radiotherapy, making wide local excision the most efficient approach [66].

Recently, Gutteridge et al. evaluated the monitoring and prognostic role of Isocitrate dehydrogenase type 1 (IDH1) and IDH2 mutations in ctDNA, which were linked to disease progression as well as to transformation into dedifferentiated chondrosarcoma [38]. According to their research, ctDNA was detected, through the application of digital PCR, in 14 out of 29 patients only prior to the onset of their treatment. In addition, the detection of ctDNA appeared in all patients with GIII and dedifferentiated disease as well as in 8 out of 17 cases with GII CS, but in no patient with early-stage GI disease. Interestingly, the ctDNA levels were found significantly decreased after surgical excision of the primary tumor, revealing their valuable role in liquid biopsy as potential biomarkers [38].

Although many in vitro trials have been demonstrated that miRNAs could be fundamental biomarkers for CS monitoring with liquid biopsy, we observed a lack of clinical studies in published bibliography. For instance, it is well established that miR-181a (a tumor promoter) is upregulated in high-grade and miR-100 (a tumor suppressor) is downregulated in CS.

Similarly, the presence of chromosomal translocations such as EWSR1-NR4A3, RBP56-NR4A3 and TCF12-NR4A3 in myxoid chondrosarcomas [67] as well as HEY1-NCOA2 and IRF2BP2-CDX1 in mesenchymal chondrosarcoma [68] are well identified in CS. However, none of these potential biomarkers have been evaluated sufficiently in the clinical setting.

The biomolecules studied for diagnosis and monitoring of bone sarcomas through the liquid biopsy technique as well as the results conducted are presented in Table 2.

Figure 2 illustrates the most common sites where bone sarcomas develop, the mutations identified in its tumor and the biomarkers investigated.

## 4. Conclusions

Modern medicine is increasingly shifting its focus on timely disease detection to maximize the therapeutic outcome for patients. Moreover, the inefficiency of penetrative procedures and inadequacy of tissue biopsies to unveil the histological and molecular signature of a tumor in its entirety has rendered the discovery of easily assessed tumor biomarkers imperative. Studies that altogether incorporated hundreds of patients have proven that evaluation of miRs, ctDNA, CTCs, and exosomes through the liquid biopsy technique can be applicable in clinical practice in order to identify the presence of a tumor, its molecular identity and the stage of the disease, as well as aid in the development of the proper therapeutic intervention. Beyond that, liquid biopsy can also contribute to the fields of treatment monitoring, patients’ follow up and prompt discovery of recurrence. Furthermore, one of the greatest clinical relevance of liquid biopsy in the clinical oncology setting consists of the application of a personalized diagnostic and monitoring tool as well as the development and administration of customized therapies based on the detected gene alterations in sarcoma patients. We believe that the identification of gene alterations, which have been detected and utilized by liquid biopsy, represents a fundamental advancement in precision medicine compared with the use of less specific and sensitive biomarkers (e.g., detection of ALP and LDH) with conventional techniques. While the relatively high cost and limited array of biomarkers represent issues that need to be further addressed, we believe that the use of liquid biopsy in modern oncology comprises a promising novel method in the fields of disease diagnosis, monitoring and treatment.

## Figures and Tables

**Table 1:** Markers studied through the liquid biopsy technique in soft tissue sarcomas, number of samples, methods utilized and results conducted.

Markers Studied	Samples Included	Methods	Results	Ref
Liposarcoma				
miR-145 miR-451	57 tissue samples and liquid biopsies	miR microarray	miR expression profiling to discriminate liposarcoma subtypes miR-145 and miR-451 behave as tumor suppressors re-expression of miR-145 and miR-451 means achievement of therapeutic goal	[12]
ctDNA cfDNA - myxoid liposarcomas: t(12;16) and TERT C228T promoter mutation - well-differentiated/de-differentiated liposarcomas: MDM2 amplifications	Patients with STS (*n* = 64) Patients with remission (*n* = 19) Healthy controls (*n* = 41) Patients with myxoid liposarcomas (*n* = 4)Patients with well-differentiated/de-differentiated liposarcomas (*n* = 5)	qRT-PCR	ctDNA detects minimal residual disease and recurrence cfDNA was specific for the monitoring of patients with myxoid liposarcomas MDM2 amplifications was not sensitive for the detection of patients with well-differentiated/de-differentiated liposarcomas	[13]
TP53 mutations in cfDNA	DDL (*n* = 21)	cfDNA isolation from plasma with double centrifugation	TP53 mutation correlates with tumor size TP53 mutant clones resistant to HDM2−antagonists	[14]
miR-3613-3p miR-4668-5p	DDL patients (*n* = 6) Healthy individuals (*n* = 4)	qRT-PCR	miR-3613-3p is a specific biomarker for DDL	[15]
**Leiomyosarcoma**				
cfDNA paired genomic DNA from resected tumors	Patients (*n* = 30) 29/30 patients with matched tumor sample 8 patients with tumor samples only	Extracted DNA was quantified using Quant-iT PicoGreen dsDNA Assay Kit	LMS ctDNA in 11/16 (69%) patients with disease progression and total tumor burden ≥ 5 cm No detected ctDNA in 16 patients with stable or low disease ctDNA levels declined after tumor resection (patient *n* = 1) ctDNA detectable after disease relapse (patient *n* = 1)	[16]
ctDNA CGP	Patients (*n* = 6)−blood samples	FoundationACT™ assay (F1ACT)	No correlation between tumor fraction or radiographic tumor volume	[17]
**Ewing sarcoma**				
exosome-associated proteins CD63 and CD81 EWS-FLI1	ES cell lines (A673, SK-N-MC and SB-KMS-KS1)	qRT-PCR (Exosomes were prepared from the cell culture supernatant)	exosomes are potential biomarkers for diagnosis of minimal residual disease	[18]
In vitro: EWS/FlI1 mRNA In vivo: inoculated xenografts with TC135 or A673 cells.	culture medium of ES cell lines plasma samples from ES cell/xenografted mice	qRT-PCR	*EWS/Fli-1* mRNA in microvesicles is a potential non-invasive biomarker	[19]
CD-99 on the surface of CTCs *EWSR1/FLI1* type 1/type 2 *EWSR1/ETS*-related gene transcripts type 1/type 9e.	Blood samples from healthy volunteers (*n* = 9) ES patients (*n* = 18)	qRT-PCR	CTCs in liquids can be used for prognostic and predictive purposes	[20]
EWSR1-FLI1 ctDNA	234 blood samples from 20 ES patients before and after multimodal treatment	droplet digital PCR	ctDNA for the monitoring of therapy response	[21]
*EWS-FLI1* DNA breakpoints	Blood samples and tissue biopsy from ES patients (*n*=5)	RT-qPCR digital PCR	*EWS-FLI1* DNA useful in monitoring: ES progression disease’s response during surgical and chemotherapeutic treatment	[22]
EWS-ETS fusion gene breakpoint ptDNA fragments	TC71 xenograft or PDX EWS 1 or EWS4 (*n* = 5) plasma DNA from ES patients (*n* = 3) patients with no detectable disease on x-rays (*n* = 2)	long range multiplex PCR droplet digital PCR	EWS-ETS fusion gene breakpoint ptDNA fragments is a specific biomarker to monitor tumor relapse	[23]
EWS-ETS transcripts	ESFT cell lines (*n* = 5)	Proteomic study of ESFT-derived sEVs from 5 ESFT cell lines	sEVs are potential prognostic biomarkers	[24]
**Rhabdomyosarcoma**				
ctDNA	ARMS patients (*n* = 7)	ULP-WGS	ctDNA was measurable in ≥ 50% of pre-treatment plasma samples from ARMS patients All patients with ctDNA specific blood sampled were positive for PAX3/FOXO1 translocation	[25]
miR-206	RMS patients (*n* = 10) cell lines (Rh30, SCMC-RM2, RD, RMS-YM, CT-TC, Rh18, Rh41	RT-qPCR	serum miR-206 expression differentiates RMS and non-RMS tumors	[26]
**Synovial sarcoma**				
SS18-SSX fusion genes from CTCs or cell-free nucleic acids	Blood sample from SS patients (*n* = 15)	nested PCR droplet digital PCR	detection of SS18-SSX fusion gene after surgery/chemotherapy/radiotherapy is a rare circumstance SS18-SSX fusion gene not sufficient for monitoring of recurrence	[27]
SS18-SSX1/2 fusion genes in CTCs	Blood samples from SS patients (*n* = 38)	nested PCR	low detection rate of SS18-SSX1/2 in circulation low sensitivity of SS18-SSX1/2 for tumor detection	[28]
miR-92b-3p	serum samples from SS patients (*n* = 12) Vs other STS patients (*n* = 24) SYO-1, HS-SY-II, and YaFuSS cell lines SS-bearing mice	RT-qPCR	serum miR-92b-3p levels were significantly higher in SS patients compared to healthy individuals *miR-92b-3p* discriminates patients with SS from the other STS patients potential clinical significance of serum *miR-92b-3p* for the monitoring of SS	[29]

**Table 2:** Parameters measured through liquid biopsy in bone sarcomas, number of samples, methods used and results.

Markers Studied	Samples Included	Methods	Results	Ref
Osteosarcoma				
ctDNA	blood samples of OS patients (*n* = 72)	NGS assays	41/72 (56.9%) patients had detectable ctDNA at the time of diagnosis. 11% (range 4.6–58%) of total cell-free DNA was ctDNA worse outcome and death associated with increased levels of ctDNA 8q gain was detected in 74.4% OS patients	[30]
mutated alterations in TP53, RB1, ATRX, DLG2, PTEN, MET, and SLC19A1 in ctDNA	blood samples OS patients (*n* = 7)	NGS assays	clinical relapse in 3 patients with germline-tumor pairs	[31]
miRNAs tumor suppressors: miR-326, miR-133b, miR-206, miR-152, miR-95–3p, miR-34b, miR-195, miR-223, miR-497 oncogenic miRNAs: miR-21, miR-196a, miR-196b, miR-195–5p and miR-199a-3p, miR-199a-5p, miR-221, miR-27a LncRNAs: TUG1, 91H UCA1, lncRNA-ATB, MALAT1	Blood samples of OS compared with health controls	RT-qPCR	potential value as serum biomarkers for detection and clinical evaluation of OS patients assessment of therapeutic interventions and responsiveness to chemotherapy correlated with systemic expansion, tumor relapse and poorer prognosis	[32]
CTCs surface-type Vimentin ezrin centromeres of chromosome 8	Blood samples of OS patients	RNA probe technology FISH to detect centromeres of chromosome 8	CTCS can be discriminated from mononuclear blood cells by detecting Vimentin High expression of ezrin in CTCs correlated significantly with the formation of distant metastases Patients with pulmonary metastases revealed higher circulating levels of CTCs compared with patients free of metastases. High CTCs levels were correlated with poorer prognosis for OS patients	[33,34,35]
extracellular vesicle (EV)-educated mesenchymal stem cells (TEMSC) which express a membrane-associated form of TGFβ	Blood samples from OS mice xenograft models	RT-qPCR	Prognostic and predictive tool in OS	[36]
Exosomes containing: miR-124, miR133a, miR-199a-3p, and miR-385	Blood samples of: 48 OS patients with poor chemotherapeutic response 45 OS patients with good chemotherapeutic response 51 healthy controls	RT-qPCR	exosomal miRNAs could be reliable circulating biomarkers to discriminate poor from good treatment responders	[37]
**Chondrosarcoma**				
IDH1 and IDH2 mutations in ctDNA	Blood samples from patients with central chondrosarcoma (*n* = 29)	digital PCR	ctDNA was detected in pretreatment samples in 14/29 patients clear reduction following surgical removal	[38]

## Abbreviations

OS	osteosarcoma
NGS	Next-generation sequencing
FISH	fluorescence in situ hybridization
CTCs	circulating tumor cells
IDH1	Isocitrate dehydrogenase type 1
IDH2	Isocitrate dehydrogenase type 2
miR	micro-RNA
ctDNA	circulating tumor DNA
cfDNA	cell-free DNA
STS	soft tissue sarcomas
CTC	circulating tumor cells
DDL	dedifferentiated liposarcoma
CGP	comprehensive genomic profiling
ptDNA	plasma tumor DNA
PDX	patient derived xenograft
ESFT	Ewing Sarcoma Family Tumors
sEVs	Small ESFT-associated extracellular vesicles
ARMS	alveolar rhabdomyosarcoma
RMS	rhabdomyosarcoma
ULP-WGS	ultralow passage whole-genome sequencing
SS	synovial sarcoma
